# Engineering Novel Lentiviral Vectors for Labelling Tumour Cells and Oncogenic Proteins

**DOI:** 10.3390/bioengineering9030091

**Published:** 2022-02-25

**Authors:** Seçkin Akgül, Carolin Offenhäuser, Anja Kordowski, Bryan W. Day

**Affiliations:** 1 Sid Faithfull Brain Cancer Laboratory, Cell and Molecular Biology Department, QIMR Berghofer Medical Research Institute, Brisbane, QLD 4006, Australia; seckin.akgul@griffithuni.edu.au (S.A.); carolin.offenhauser@qimrberghofer.edu.au (C.O.); anja.kordowski@qimrberghofer.edu.au (A.K.); 2 School of Medicine and Dentistry, Griffith University, Gold Coast, QLD 4215, Australia; 3 School of Medicine, University of Queensland, St Lucia, QLD 4072, Australia; 4 School of Biomedical Sciences, Faculty of Health, Queensland University of Technology, Brisbane, QLD 4059, Australia

**Keywords:** lentivirus, plasmid, lentiviral particle, site-directed mutagenesis, molecular cloning, genetic engineering, tumour heterogeneity

## Abstract

Lentiviral vectors are unique and highly efficient genetic tools to incorporate genetic materials into the genome of a variety of cells whilst conserving biosafety. Their rapid acceptance made it necessary to improve existing protocols, including molecular engineering and cloning, production of purified lentiviral particles, and efficient infection of target cells. In addition to traditional protocols, which can be time-consuming, several biotechnology companies are providing scientists with commercially available lentiviral constructs and particles. However, these constructs are limited by their original form, tend to be costly, and lack the flexibility to re-engineer based on the ever-changing needs of scientific projects. Therefore, the current study organizes the existing methods and integrates them with novel ideas to establish a protocol that is simple and efficient to implement. In this study we, (i) generated an innovative site-directed nucleotide attachment/replacement and DNA insertion method using unique PCR primers, (ii) improved traditional methods by integrating plasmid clarification steps, (iii) utilized endogenous mRNA as a resource to construct new lentiviruses, and (iv) identified an existing purification method and incorporated it into an organized workflow to produce high-yield lentiviral particle collection. Finally, (v) we verified and demonstrated the functional validity of our methods using an infection strategy.

## 1. Introduction

Lentiviral vectors are widely used for delivery of complex genetic structures, most commonly including miRNAs [1], siRNAs or shRNAs [2], fluorescent proteins [3], oncogenes, tumour suppressor genes, and stem-cell associated genes [4]. Furthermore, lentiviral technology is used in generating transgenic animals, in vivo imaging, cell-lineage tracking, site-directed gene editing, and gene therapy [5,6]. There are several advantages of using lentiviruses as expression vectors. One of the major benefits is stable long-term expression in a wide variety of dividing and non-dividing cell types, including those that are challenging to transfect with traditional transfection methods [6,7]. While other vector systems (e.g., oncogenic retroviruses and expression plasmids) have been reported to be silenced after several cell passages, expression by lentiviral gene transfer remains sustained [8,9]. Furthermore, the expression levels of these transgenes have a relatively narrow range that is mostly determined by the number of copies delivered to each cell [6]. This feature makes it suitable to design experiments with different types of cell lines. Thirdly, the separation of cis-acting genes (genes that allow transfer of viral genome to target cells) from trans-acting genes (genes that encode viral proteins) provides significant biosafety measures that limit cell infection to a controlled single round. Moreover, lentiviruses are considered to have low immunogenicity and toxicity [10]. Lastly, lentiviral woodchuck hepatitis virus posttranscriptional regulatory element (WPRE) promotes post-transcriptional polyadenylation that provides RNA processing and nuclear export of the viral and transgene RNA resulting in increased transgene expression [11,12].

Even though lentiviral technology is one of the most preferred methods, molecular engineering of these vectors prior to their use has several challenges that cost researchers an excessive amount of time and money. These challenges include, (i) production of a relatively long gene (e.g., 10 mb) and its insertion into a lentiviral backbone; (ii) finding unique restriction sites to extract and ligate DNA fragments; (iii) lack of restriction sites in inserts and/or vectors; (iv) GC-rich sequences in primers that impede a proper polymerase chain reaction (PCR); (v) repeated sequences in transgene that leads to incorrect insert in the lentiviral plasmid; (vi) purity, accuracy, and quality of starting material, particularly of those that have been transferred from another laboratory.

In addition to various obstacles in molecular engineering of lentiviral constructs, isolation of viral particles in a laboratory environment has added challenges, including efficient handling of virus-containing supernatant, eliminating virus-producing cells and cell debris, maintaining a high titer of functional viral particles, minimizing losses during the clarification and purification procedures, and ensuring affordability [13,14]. Currently, several protocols are being used in the laboratories with distinct advantages and disadvantages [13]. The main techniques are anion-exchange chromatography [15], affinity chromatography [16], size-exclusion chromatography [17], and ultracentrifugation [18]. However, production of high-titer, highly pure, and functional lentivirus with simple, scalable, and economical methods with rapid processing of large volumes of virus-containing supernatant is still challenging [14,19].

In the current study, we designed simple and effective laboratory techniques that utilize lentiviral particles and endogenous mRNA molecules to incorporate DNA sequences into the genome of the cultured mammalian cells. We revised the current knowledge and protocols and improved them to circumvent the challenges described above. Our methodology relies on novel PCR techniques that can amplify long DNA fragments, molecular cloning strategies with unique primers that can induce site-directed mutagenesis and site-directed DNA integration and also commercially affordable purification methods that ensure high-yield viral particles with efficient infectivity. It is worth noting that all these techniques can be performed within a short time without causing any financial burden.

## 2. Materials and Methods

### 2.1. Primary Cell Culture

Glioblastoma primary cell lines and culturing techniques were described previously [20,21] and followed the basic protocol outlined by Pollard and colleagues [22]. Briefly, cell culture media included KnockOut™ DMEM/F-12, GlutaMAX™ Supplement, StemPro™ Neural Supplement, Recombinant Human EGF, Recombinant Human FGFb, and Penicillin/Streptomycin (Thermo Fisher Scientific, Scoresby, VIC, Australia). Cells were cultured on flasks coated with Basement Membrane Matrigel^®^ Matrix. Cell passaging was done by detaching the cells from the flask surface using Accutase^®^ solution (Sigma Aldrich, North Ryde, NSW, Australia, cat. H9268).

### 2.2. Western Blotting

Cells were lysed in ice-cold Pierce RIPA buffer (Thermo Fisher Scientific, Scoresby, VIC, Australia) containing cOmplete Protease Inhibitor Cocktail and PhosSTOP (both from Merck, Bayswater, VIC, Australia). Post-nuclear supernatants were collected and protein content determined by Bio-Rad protein assay (Bio-Rad Laboratories, South Granville, NSW, Australia). Proteins were separated by SDS-PAGE under reducing conditions and protein expression of EphA3, HA-tag, and β-actin was determined by western blot analysis.

### 2.3. Flow Cytometry

Cell surface expression of EphA3 was analysed by flow cytometry using an in-house anti-EphA3 monoclonal antibody termed IIIA4 (5 ug/mL). Mouse IgG1 isotype control antibody was used to determine background staining. Cells were detached from the cell culture flask using 5 mM EDTA in PBS and resuspended in FACS buffer (4% FCS, 0.2% human IgG in PBS). After blocking, cells were incubated with the primary antibody in FACS buffer for 30 min on ice, washed and incubated with secondary Alexa Fluor 647-conjugated anti-mouse antibodies in FACS buffer for 20 min on ice in the dark, then washed and fixed in 1% PFA/PBS. EphA3 expression levels were determined using an LSR Fortessa flow cytometer (BD Biosciences, Macquarie Park, Australia) and data analysis was performed using FlowJo (FlowJo, LCC, Ashland, OR, USA) software.

### 2.4. Materials

- pLVX-AmCyan1-C1 plasmid (Clontech, Orchard Parkway, San Jose, CA, USA, cat. 632557)
- pE2. Crimson plasmid (Clontech, cat. 632553)
- pZsYellow plasmid (Clontech, cat. 632443)
- Q5 Hot Start High-Fidelity 2X Master Mix (New England BioLabs, Notting Hill, VIC, Australia, cat. M0494S)
- XhoI restriction enzyme (New England BioLabs, cat. R0146S)
- EcoRI-HF restriction enzyme (New England BioLabs, cat. R3101S)
- T4 DNA Ligase (New England BioLabs, cat. M0202S)
- NEB 5-alpha Competent E. coli (High efficiency) (New England BioLabs, cat. C2987H)
- DMEM (high glucose, pyruvate) (Thermo Fisher Scientific, Scoresby, VIC, Australia, cat. 11995073)
- 10% FBS (Thermo Fisher Scientific, cat. 10099141)
- Lipofectamine™ 3000 Transfection Reagent (Thermo Fisher Scientific, cat. L3000015)
- Opti-MEM Reduced Serum Medium (Thermo Fisher Scientific, cat. 31985088)
- 0.45 µm Syringe Filter Unit (Merck, Bayswater, VIC, Australia, cat. SLHVO33RS)
- PEG Virus Precipitation Kit (Abcam, Melbourne, VIC, Australia, cat. Ab102538)
- Hexadimethrine bromide (Polybrene) (Sigma Aldrich, North Ryde, NSW, Australia, cat. H9268)
- KnockOut™ DMEM/F-12 (Thermo Fisher Scientific, cat 12660-012)
- GlutaMAX™ Supplement (Thermo Fisher Scientific, cat. A12860-01)
- StemPro™ Neural Supplement (Thermo Fisher Scientific, cat. A10508-01)
- Recombinant Human EGF (Thermo Fisher Scientific, cat. PHG0314)
- Recombinant Human FGFb (Thermo Fisher Scientific, cat. PHG0024)
- Penicillin/Streptomycin (Thermo Fisher Scientific, cat. 15140122)
- Basement Membrane Matrigel^®^ Matrix (Corning, Mulgrave, VIC, Australia, cat. 354234)
- Accutase^®^ solution (Sigma Aldrich, cat. A6964)
- HA-tag antibody (Cell Signaling Technology (New England Biolabs), Notting Hill, VIC, Australia, cat. 3724)
- EphA3 antibody (for FACS and western blotting) (Invitrogen, Thermo Fisher Scientific, cat. 37-3200)
- β-actin antibody (Cell Signaling Technology (New England BioLabs), cat. 3700)
- Mouse IgG1 isotype control antibody (Cell Signaling Technologies (New England BioLabs), cat. 5415S)
- Alexa Fluor 647 (Thermo Fisher Scientific, cat. A-31571)

## 3. Results

### 3.1. Site-Directed Amplification-Restriction-Ligation Strategy for Exchanging DNA Fragments between Vectors

**Figure 1:**
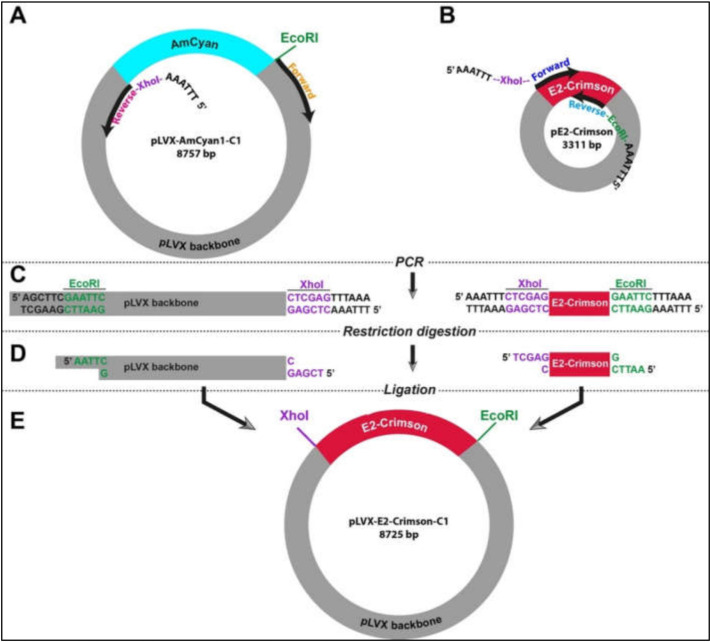
The outline of the molecular cloning strategy to engineer novel lentiviral plasmids (**A**,**B**). Specific primers were designed to incorporate restriction sites in flanking regions of both vector (pLVX) and insert (E2-Crimson) (**C**). High-fidelity polymerase chain reaction enabled the production of linear DNA fragments (**D**). Restriction digestion with XhoI and EcoRI resulted in complementary sticky ends in the vector and insert (**E**). Ligation reaction allowed the specific fusion of two DNA fragments yielding a new lentiviral plasmid that carry the E2-Crimson fluorescent protein gene.

In the absence of flanking restriction sites, it can be confounding to prepare an empty vector and a DNA insert that can be used in a ligation reaction. Therefore, we designed a strategy using simple laboratory techniques to facilitate the exchange of DNA fragments between different vectors. This strategy relies on introducing new restriction sites at any of the 5′ and/or 3′ end of DNA fragments that can be fused by a ligation reaction (Figure S1). Two PCR experiments were designed in parallel in order to create an empty vector (pLVX) and the insert (E2-Crimson). In the case of the vector, pLVX-AmCyan1-C1 was used as a template (Table S2). As opposed to traditional PCR reactions where two primers amplify a DNA region of 100 kb-2 mb in size, the specific primers for amplification of the vector were approximately 8 kb apart from each other (Figure 1A). This strategy required using a high-fidelity DNA polymerase (Q5 High-Fidelity DNA Polymerase). E2-Crimson insert (700 bp) was amplified from pE2-Crimson bacterial plasmid, which is a prokaryotic expression vector and does not have the lentiviral properties (Figure 1B and Table S3). Both amplification reactions were performed using specific primers that contained XhoI (CTC GAG) or EcoRI (GAA TTC) that do not have complementary sequences on the insert and vector (Figure 1C and Table S1). Furthermore, a hexamer (AAA TTT or TTT AAA) was added to the 5′ end of the primers in order to create a suitable DNA binding space for the restriction enzymes. After restriction digestion reactions with XhoI and EcoRI (Figure 1D and Table S1), DNA fragments were specifically fused to each other in a T4 DNA ligation reaction (Figure 1E). This final ligation produced a new plasmid (pLVX-E2. Crimson-C1) that can be used in producing lentiviral particles that will allow the expression of E2-Crimson fluorescent protein under the CMV promoter in mammalian cells.

It is important to note that two DNA purification steps were incorporated in the procedures described above. The first one was performed after the PCR experiment before proceeding to the restriction digestion reaction. The second one was performed after the restriction digestion reaction before the ligation reaction (Figure S1). Both purifying procedures were performed in order to remove the excess salt, unused dNTPs, and glycerol that can reduce the efficiency of these enzymatic reactions.

### 3.2. Engineering Lentiviral Constructs with Fluorescent Protein Genes

**Figure 2:**
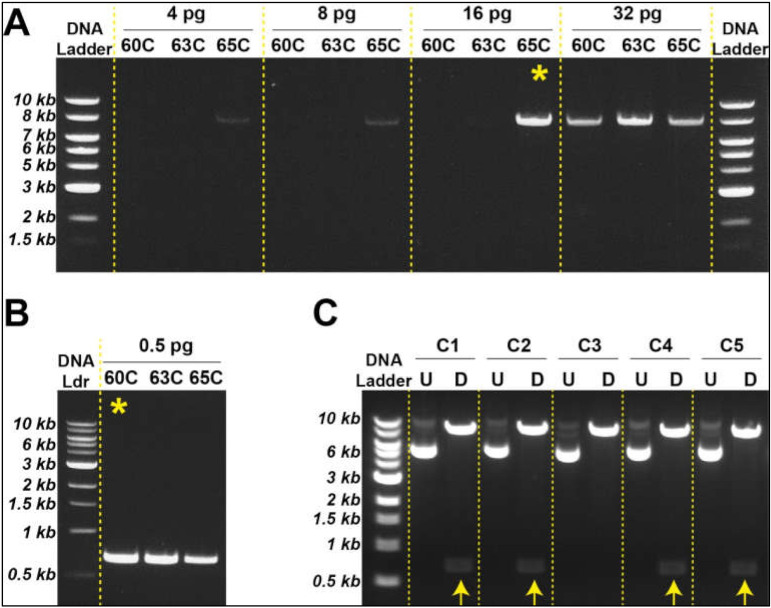
Generation of lentiviral plasmids with different fluorescent protein genes (**A**). Optimum template DNA and annealing temperature is determined to synthesize empty vector (pLVX). The most advantageous tested condition is indicated with a yellow asterisk (**B**). Minimal DNA template was used to synthesize insert (E2-Crimson). The most advantageous tested condition is indicated with a yellow asterisk (**C**). Restriction digestion with XhoI and EcoRI identified the newly engineered plasmids with correct DNA sizes (indicated by yellow arrows).

Based on the strategy described above, we generated an empty pLVX vector using different amounts of template plasmid DNA (i.e., pLVX-AmCyan1-C1). Our results suggested that specific DNA amplification can be achieved with as low as 16 pg template DNA at 65 °C (Figure 2A); however, these two parameters can vary from reaction to reaction and should be tested individually. We could similarly amplify the E2-Crimson gene construct using only 500 ng template DNA at 60 °C (Figure 2B). Once specific DNA fragments were digested using XhoI and EcoRI, sticky ends were ligated using T4 DNA ligase (Figure 1D,E) followed by bacterial transformation using heat shock technique (Figure S2A,B). Newly engineered plasmids were extracted from transformed bacteria by miniprep and subjected to restriction digestion with XhoI and EcoRI in order to determine the nature of the ligated DNA products. Four out of five bacteria clones produced the expected sizes of DNA fragments (i.e., 8 kb and 0.7 kb) (Figure 2C). Two of these clones were sequenced to ensure that the insert did not have any mutations that might be introduced during the PCR amplification step (Tables S5 and S6). Once sequencing data confirmed the accurate insertion of the E2-Crimson gene into pLVX vector, we amplified this plasmid construct using midiprep. We repeated this strategy to transfer ZsYellow fluorescent protein gene construct from pZsYellow (Table S4) into pLVX backbone.

### 3.3. Cloning Membrane Receptor Genes Using Tumour Cell Line mRNA

**Figure 3:**
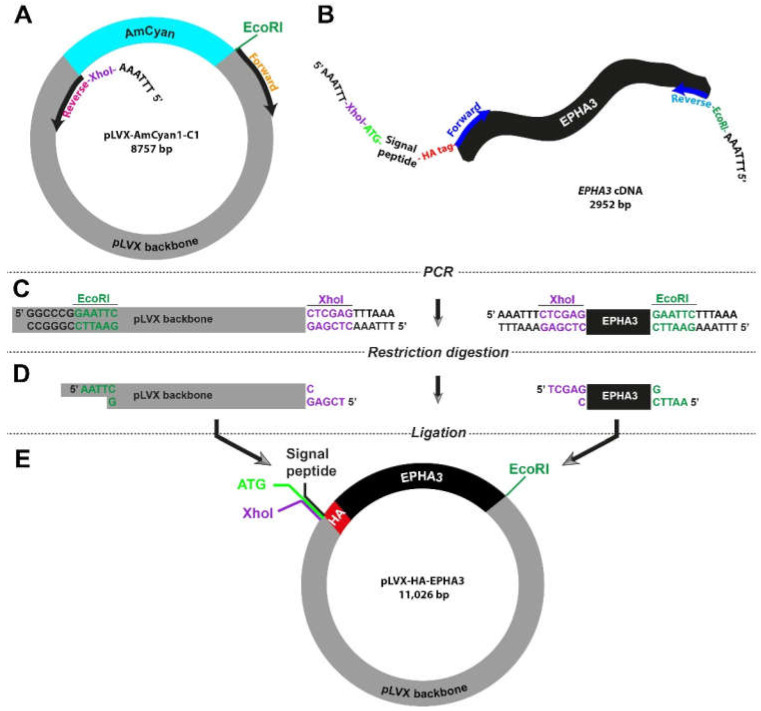
Outline of the molecular cloning strategy to introduce small peptides in wild type genes (**A**,**B**). Specific primers were designed to incorporate restriction sites in flanking regions of both vector (pLVX) and insert (*EPHA3*). HA-tag is added after the 20-aa signal peptide of *EPHA3* gene (**C**). High-fidelity polymerase chain reaction enabled production of linear DNA fragments (**D**). Restriction digestion with XhoI and EcoRI resulted in complementary sticky ends in the vector and insert (**E**). Ligation reaction allowed specific fusion of two DNA fragments, yielding a new lentiviral plasmid that carries the HA-tagged *EPHA3* gene.

We expanded our molecular cloning strategy to address situations where a gene of interest is not available in a vector. The EphA3 receptor tyrosine kinase has previously been reported to be over expressed and functional in human cancers [23]. For this purpose, we used a transmembrane receptor gene, *EPHA3*, as a model, and the QCell human glioblastoma cell line HW1 as the source of the gene [24,25]. Based upon our previous studies, we had determined that HW1 cells express high EphA3 levels [20,21,24,26]. We followed a two-step amplification process: (a) HW1 cDNA pool was prepared with olidoDT primers in a reverse transcriptase reaction (b) Purified cDNA was further amplified using *EPHA3*-specific primers that carry restriction digestion sites in their flanking regions (Figure 3). These specific primers also allowed us to insert HA-tag into the 5′ end of the *EPHA3* gene (described in the next section).

### 3.4. Site-Directed Amplification-Restriction-Ligation Strategy for Insertion of Short Peptide Sequences into Wild-Type Genes

Small peptides, including HA-tag, His-tag, and Flag-tag, are widely used as molecular biology tools to label proteins in western blotting and immunofluorescence experiments. Insertion of these peptide sequences into flanking regions of studied proteins can be challenging and time-consuming. Therefore, we decided to test whether our high-fidelity Amplification-Restriction-Ligation strategy can be utilized in engineering constructs with a labelling tag. For proof-of-concept purposes, we picked HA-tag (Table 1) to be inserted into the 5′ region of the *EPHA3* gene.

As the vector, we used the pLVX-AmCyan1-C1 plasmid and designed the primers so that the AmCyan1 sequence would be excluded from the final product (Figure 3A). The key feature of the forward primer for the *EPHA3* gene extracted from the HW1 cell line was the fact that it also included the DNA sequence of HA-tag, which is 27 bp long (Figure 3B and Table S1). It is important to note that proteins that are targeted to the endoplasmic reticulum and eventually destined to be localized in the cell membrane carry a “signal peptide” in their N-terminus. As such, there is a 20-amino-acid-long signal peptide at the N-terminus of the EphA3 protein, which is removed through a post-translational modification mechanism producing a mature protein that translocates in the cell membrane. Therefore, we designed primers so that the HA-tag is inserted after this signal peptide to prevent cleavage via this post-translational modification process (Figure 3B and Table S1). These specific primers also contained XhoI (CTC GAG) or EcoRI (GAA TTC) in order to fuse the final PCR products. Lastly, all primers were accompanied by an additional “AAA TTT” or “TTT AAA” site that would allow the restriction enzymes to engage with the DNA. This strategy required the utilization of a notably long forward primer (114 bases) for the amplification of the insert (i.e., HA-tagged *EPHA3* gene). The remaining steps were similar to the strategy that we utilized previously (Figure 1C and Figure 3C,D). The final lentiviral plasmid, pLVX-HA-EPHA3, is as big as 11 kb and harbor the HA-tagged *EPHA3* gene under the CMV promoter (Figure 3E).

### 3.5. Engineering Lentiviral Constructs with HA-Tagged Genes

**Figure 4:**
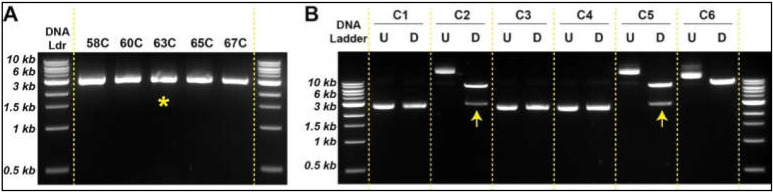
Generation of new lentiviral plasmids allows HA-tagged proteins (**A**). Optimum annealing temperature is determined to synthesize insert (HA.EPHA3). The most advantageous tested condition is indicated with a yellow asterisk (**B**). Restriction digestion with XhoI and EcoRI identified the newly engineered pLVX.HA.EPHA3 plasmids with correct DNA sizes (indicated by an arrow). Abbreviations: U: undigested DNA; D: digested DNA.

Using the strategy outlined in Figure 3, we amplified both the empty vector (pLVX) and the insert (HA.EPHA3). Preparation of pLVX empty vector was the same as the protocol we followed in Figure 2A. Since the coding sequence of the *EPHA3* is rather a long DNA fragment (2949 bp), we tested five different annealing temperatures (58 °C, 60 °C, 63 °C, 65 °C, and 67 °C) using HW1 cDNA as a template (Figure 4A). Though there was no significant difference between these five temperatures in terms of amplification yield, we chose 63 °C for consistency with other PCR experiments. Furthermore, the notable differences in length (114 bases forward, 30 bases reverse) and Tm (95 °C forward vs. 63 °C reverse) of the paired primers did not seem to affect the amplification of the targeted DNA sequences negatively (Figure 4A and Table S1).

Once specific DNA fragments were digested using XhoI and EcoRI, sticky ends were ligated using T4 DNA ligase (Figure 3D,E) followed by bacterial transformation using heat shock technique. Newly engineered plasmids were extracted from transformed bacteria by miniprep and subjected to restriction digestion with XhoI and EcoRI to determine the nature of the ligated DNA products. Two out of six bacteria clones produced the expected sizes of DNA fragments (i.e., 8.2 kb and 2.9 kb) (Figure 4B), suggesting that the creation of the correct plasmid using two large DNA sequences is relatively less efficient. Sequencing data confirmed the accurate insertion of the HA-EPHA3 gene in the pLVX lentiviral plasmid. We then purified this plasmid construct using midiprep.

### 3.6. Preparation and Purification of Lentiviral Particles

High cost of commercially available lentiviral particles encourages researchers to produce them in a laboratory environment. However, there are several challenges in preparing lentiviral particles, including low viral particle yield, extensive protocols, contamination, and suboptimal infection efficiency. Furthermore, using the supernatant collected from virus-producing cell lines (e.g., HEK293T cells) without a purification procedure interferes with the molecular mechanisms of some sensitive cell lines, including stem cells and primary tumour lines, which greatly affects their proliferation, differentiation, and self-renewal. We, therefore, reviewed publicly available protocols and biotechnology products to generate a simple protocol to prepare high-yield viral particles (Section 3.6.1, Section 3.6.2, Section 3.6.3). This protocol allowed us to prepare purified lentiviral particles easily and in a cost-effective manner.

#### 3.6.1. Transfection of HEK293T Cells with Lentiviral Plasmids

1. Five million HEK293T cells were cultured in T75 flasks using DMEM (high glucose, pyruvate) with 10% FBS for 16 h in a CO_2_ incubator.
2. Lipofectamine™ 3000 Transfection Reagent was used for transfection procedure. Briefly, Solution-1 was prepared using 45 uL Lipofectamine-3000 and 3 mL Opti-MEM Reduced Serum Medium. Solution-2 was prepared using a 15 ug lentiviral plasmid mixture (see Table 2), 30 uL P3000 reagent, and 3 mL Opti-MEM.
3. Solution-1 and Solution-2 was mixed gently and incubated at room temperature for 15 min for DNA-lipid complex to form.
4. DMEM medium of HEK293T cells is discarded; cells were gently washed once with PBS.
5. 6 mL of DNA-lipid complex was added through the wall of the flask gently. Cells were incubated with this complex for 5 h in the incubator.
6. DNA-lipid complex was discarded gently. No PBS washing was applied.
7. 12 mL of DMEM+FBS medium was added slowly through the wall of the flask in order not to detach cells that were already stressed by the transfection mixture. This day was then noted as “day-0”.

#### 3.6.2. Collection of Lentiviral Particles

1. Conditioned DMEM-FBS medium that contained lentiviral particles was collected on “day-3” and filtered using a 0.45 µm Syringe Filter Unit. It is important not to use smaller filter pores in order to maintain the integrity of the viral particles.
2. Filtered supernatant was stored at −80 °C.
3. 12 mL of fresh DMEM-FBS was added into the T75 flask, and cells were put back to incubation.
4. Steps 1–3 was repeated on “day-5” and “day-7”. After the last collection on day 7, HEK293T cells were discarded according to standard biosafety procedures.
5. Filtered supernatant from three different collection periods was then combined, which was approximately 30 mL in total.

#### 3.6.3. Purification of Lentiviral Particles

1. 2.5 mL polyethylene glycol (PEG) of PEG Virus Precipitation Kit was added to every 10 mL of viral supernatant and mixed gently.
2. PEG + supernatant mixture was kept in a rotator at 4 °C overnight. Rigorous shaking was avoided.
3. Mixture was centrifuged at 3200× *g* for 30 min at 4 °C. The supernatant was then discarded. The white pellet contained the viral particles.
4. Viral particles were reconstituted using 300–350 μL re-suspension buffer. Viral particles can be stored at 80 °C for future use.

#### 3.6.4. Infection of Tumour Cells with Lentiviral Particles

1. Three million tumour cells were cultured in a 6-well plate (500,000 cells per well) and incubated for 16 h in a CO_2_ incubator.
2. A mixture of lentiviral infection mixture was prepared according to Table 3 below.
3. Cell culture media was aspirated from the 6-well plate and 2 mL of lentiviral infection mixture was added into each well.
4. Cells were then centrifuged at 800× *g* for 30 min at room temperature and put back into a CO_2_ incubator.
5. After three days of incubation, the lentiviral infection mixture was replaced with fresh medium or cells were passaged based on their confluency.
6. Once infected cells recovered, selection antibiotics (e.g., puromycin, blasticidin, etc.) were used to remove uninfected cells (antibiotic sensitivities of the glioblastoma primary cell lines are described by Stringer et al. and can be found in QCell) [24,25].

Using the same lentiviral preparation and purification method, we also produced viral particles from pLenti-CMV V5-LUC Blas plasmid to overexpress a firefly luciferase gene [27]. Subsequently, several glioblastoma primary cell lines were successfully labelled with luciferase gene, resulting in stable overexpression of this gene (Table S7). Notably, no FACS sorting methods were needed to enrich the infected cell fraction, as a high infection rate was already achieved.

### 3.7. The Utilization of Novel Lentiviral Particles in Cell Tracing and Protein Labelling

**Figure 5:**
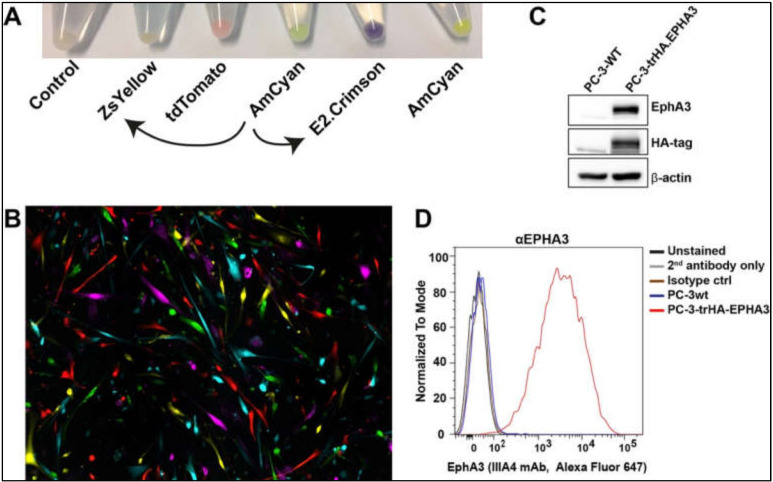
Newly designed lentiviral particles are used in cell tracing and protein labelling (**A**). Lentiviral particles allowed expression of E2. Crimson and ZsYellow fluorescent proteins. Curved arrows indicate that pLVX-E2. Crimson-C1 and pLVX-ZsYellow-C1 lentiviral plasmids were prepared by using pLVX-AmCyan1-C1 as a vector backbone (**B**). Chimeric expression of five different fluorescent proteins in tumour cells that are infected with lentiviral particles (**C**). Lentiviral particles allowed expression of HA-tagged EPHA3 protein in PC-3 cells. The left lane is wild type (wt) PC-3 cells with undetectable EPHA3 and HA-tag expression. The right lane is PC-3 cells infected with HA.EPHA3 lentiviral particles. β-actin was used as a loading control (**D**). Cell membrane localisation of HA-tagged EPHA3 protein was tested by FACS analysis of nonpermeabilized cells. EPHA3 antibody successfully detected the recombinant EPHA3 protein.

Once we prepared the lentiviral particles following the protocols outlined above, we tested their function in different tumour cell lines. Firstly, viral particles produced from the newly engineered pLVX-E2-Crimson-C1 and pLVX-ZsYellow-C1 lentiviral plasmids were used to infect a human primary glioblastoma cell line [20]. The efficiency of the viral particles was evident in cell pellets after antibiotic selection (Figure 5A). Using the commercially available lentiviruses (pLVX-tDTomato-C1, pLVX-AmCyan1-C1, and pLVX-ZsGreen-C1), we were able to label five different tumour cell clones distinctively (Figure 5B).

We then infected a prostate cancer cell line, PC-3, to determine the expression of the recombinant HA-*EPHA3* gene (Figure 5C,D). Wild type (wt) PC-3 cells have little to no EphA3 expression (Figure 5C). However, upon infection with HA.EPHA3 lentiviral particles, PC-3 had robust EphA3 expression, which was detected by both EphA3 and HA-tag antibodies in a western blotting experiment (Figure 5C). Flow cytometric analyses further validated our data, showing cell membrane localization of the recombinant EphA3 protein. Membrane localization did not appear to be affected by the incorporation of the HA-tag, as the EphA3 antibody was able to detect the surface EphA3 protein in nonpermeabilized live cells (Figure 5D). This FACS analysis also confirmed the N-terminal localization of the HA-tag, as HA-tag antibodies had access only to the N-terminus of the recombinant EphA3 protein in nonpermeabilized PC-3 cells.

These results altogether suggested that the newly engineered lentiviruses were functional and could be used to label cells and proteins for use in several applications, including protein pull-down, chromatin immunoprecipitation, ELISA, cell tracing, and subcellular localization studies.

## 4. Discussion

Here we describe a relatively straightforward procedure for DNA delivery to mammalian cells using lentiviral particles in vitro. This extended protocol is a combination of existing methods with innovative techniques of molecular engineering of lentiviral plasmids, production and purification of lentiviral particles, and infection of tumour cells lines. Our goal was to reduce the financial burden of acquiring new lentiviral plasmids or particles, expedite the time that is required for molecular cloning experiments, and provide researchers with a versatile system that can be utilized based on the needs of their projects. We chose lentiviral vector technology due to its advantages over traditional vectors, including efficient gene delivery even in non-dividing or hard-to-transfect primary cell lines, sustained gene expression after numerous passages, significant biosafety measures that limit cell infection, and low immunogenicity and toxicity.

The site-directed Amplification-Restriction-Ligation strategy that we established here relies on designing primers that allow insertion of restriction enzyme sites, start codon, and long DNA sequences (e.g., HA-tag) into the 5′- or 3′-end of genes. By amplifying a lentiviral plasmid in an “inside-out” manner, we were able to generate empty lentiviral plasmids that can be used for a variety of purposes, including exchanging a fluorescent protein gene with a different colour and replacing a blunt-end restriction site with a sticky-end restriction site. Although not demonstrated in this study, the same strategy can be used for site-directed mutagenesis as well. PCR amplification of both insert and vector allowed us to create specific DNA products, which can be easily purified using simple DNA purification kits. This strategy not only increased the efficiency of the downstream procedures (e.g., restriction digestion and DNA ligation reactions), it also allows us to circumvent tedious extraction of DNA from agarose gel.

Several sensitive cell lines, including stem cells and primary tumour cell lines, require special culture media and growth factors, and they are negatively affected by differentiation signals, including FBS. Viral particle-containing supernatant collected from HEK293T cells contain regular DMEM and also 10% FBS. Furthermore, there is a build-up of metabolic side-products that can have cytotoxic effects. Therefore, using purified lentiviral particles has substantial benefits during the infection of these delicate cell lines. However, commercially available viral particles or the majority of the purification kits are costly and time consuming to apply. The purification strategy that we adopted and improved in the current study has significant advantages to overcome these cytotoxicity or differentiation issues. However, we recommend adjusting viral titration for each cell line to achieve the highest infection rate while minimising cytotoxicity.

We demonstrated a strategy to generate lentiviral constructs that carry different fluorescent protein genes and additionally a well-known oncogene (i.e., *EPHA3*) labelled with HA-tag. Different fluorescent gene constructs can be utilized in labelling diverse tumour clones prior to reconstruction of polyclonal cell lines. HA-tagged *EPHA3* will allow researchers to study this oncogene in greater detail, including its membrane localization and function in different conditions, such as growth factors, inhibitors, antibodies, chemotherapy, and radiotherapy. Finally, it should be noted that the strategy we present is flexible enough to be tailored according to the specifics of many other scientific projects based on the requirements of the researcher.

## Figures and Tables

**Table 1:** DNA and amino-acid sequences of HA-tag.

Human Influenza Hemagglutinin (HA) Tag	
DNA sequence	5′ TAC CCA TAC GAT GTT CCA GAT TAC GCT 3′
Amino-acid sequence (3-letter) Amino-acid sequence (1-letter)	Tyr Pro Tyr Asp Val Pro Asp Tyr Ala YPYDVPDYA

**Table 2:** General protocol for preparation of Lipofectamine transfection mixtures †.

Vector Type	Vector Name	Vector for a T75 Flask	P3000-Reagent (2 μL/μg DNA)	Lipofectamine-3000 (3:1 Lipo:DNA Ratio)
Experimental vector	pLVX-E2-Crimson-C1 *	7.5 μg	15 μL	22.5 μL
Envelop vector	pCMV-VSV-G	3.3 μg	6.6 μL	9.9 μL
Packaging vector	pCMV-dR8.2 dvpr	4.2 μg	8.4 μL	12.6 μL
TOTAL		15 μg	30 μL	45 μL

† The numbers indicated should be multiplied uniformly based on the needs of the experiments.* Other lentiviral plasmids (e.g., pLVX-ZsYellow-C1 or pLVX-HA.EPHA3) were also used as experimental vectors during this study.

**Table 3:** A general protocol for preparation of lentiviral infection mixture †.

Reagent	Volume
Appropriate cell culture media * Hexadimethrine bromide (Polybrene) (4 mg/mL in water)	12 mL 24 μL
Reconstituted lentiviral particles (prepared in Section 3.6.3 above)	100 μL

† The numbers indicated should be multiplied uniformly based on the needs of the experiments.* For QCell glioblastoma primary cell lines, culturing techniques were described previously [20].

## Data Availability

The data presented in this study are available in “Engineering novel lentiviral vectors for labelling tumour cells and oncogenic proteins.” Bioengineering 2022, 9, 91. https://doi.org/10.3390/bioengineering9030091.

## Supplementary Materials

The following supporting information can be downloaded at: https://www.mdpi.com/article/10.3390/bioengineering9030091/s1, Table S1: DNA sequences of primers used in amplification of vectors and inserts; Table S2: DNA sequence of pLVX-AmCyan1-C1 lentiviral expression plasmid, Table S3: DNA sequence of pE2-Crimson prokaryotic expression plasmid, Table S4: DNA sequence of pZsYellow prokaryotic expression plasmid, Table S5: DNA alignment of the theorised (Query) and engineered (Sbjct) sequences of pLVX-E2. Crimson-C1 plasmid, Table S6: DNA alignment of the theorised (Query) and engineered (Sbjct) sequences of pLVX-E2. Crimson-C1 plasmid, Table S7. Primary glioblastoma cell lines that were successfully infected with lentiviral particles, Figure S1: Outline of High-Fidelity Amplification-Restriction-Ligation strategy, Figure S2: Heat shock technique is used to transform the competent bacteria with newly engineered pLVX-E2-Crimson-C1 lentiviral constructs.
