# Supplementary material for: Engineering Novel Lentiviral Vectors for Labelling Tumour Cells and Oncogenic Proteins
Source: Bioengineering (Basel). 2022 Feb 25;9(3):91. doi: 10.3390/bioengineering9030091 (PMC8945451; doi:10.3390/bioengineering9030091)
Supplement: Supplementary file 1 [file bioengineering-09-00091-s001.zip › bioengineering-1602904-supplementary.pdf]

Supplementary Material

Engineering novel lentiviral vectors in labelling tumour cells and oncogenic proteins

Table S1. DNA sequences of primers used in amplification of vectors and inserts.

Primer name	PCR product	Sequence (5′-3′)	Length	Tm (~) *	GC content
Forward-1	Empty pLVX vector	ACA AGG GCG GCA ACA GCG TG	20 bp	65°C	65%
Reverse-1	Empty pLVX vector	TTT AAA CTC GAG GGT GGC GAC CGG TAG ATC CT	32 bp	69°C	53%
Forward-2	*E2-Crimson* gene	AAA TTT CTC GAG ATG GAT AGC ACT GAG AAC GTC	33 bp	63°C	42%
Reverse-2	*E2-Crimson* gene	TTT AAA GAA TTC CTA CTG GAA CAG GTG GTG GCG	33 bp	65°C	46%
Forward-3	*ZsYellow* gene	AAA TTT CTC GAG ATG GCT CAT TCA AAG CAC GGT	33 bp	66°C	42%
Reverse-3	*ZsYellow* gene	TTT AAA GAA TTC TCA GGC CAA GGC AGA AGG GAA	33 bp	65°C	42%
Forward-4	HA-tagged *EPHA3* gene	AAA TTT CTC GAG ATG GAT TGT CAG CTC TCC ATC CTC CTC CTT CTC AGC TGC TCT GTT CTC GAC AGC TTC GGG TAC CCA TAC GAT GTT CCA GAT TAC GCT GAA CTG ATT CCG CAG	114 bp	95°C	50%
Reverse-4	HA-tagged *EPHA3* gene	TTT AAA GAA TTC TTA CAC GGG AAC TGG GCC	30 bp	63°C	43%

Extra space for restriction enzymes
XhoI restriction site (C▾TCGAG)
EcoRI restriction site (G▾AATTC)
START codon (ATG)
Signal peptide
HA-tag
* Tm values were calculated by using ApE (A Plasmid Editor) software.

Table S2. DNA sequence of pLVX-AmCyan1-C1 lentiviral expression plasmid. Functional regions are highlighted with different colours. Note the binding sites of Forward-1 (Line-28) and Reverse-1 (Line-23) primers.

tggaagggctaattcactcccaaagaagacaagatatccttgatctgtggatctaccacacacaaggctacttccctgattagcagaactacacaccagggccaggggtcagatatccactgacct
ttggatggtgctacaagctagtaccagttgagccagataaggtagaagaggccaataaaggagagaacaccagcttgttacaccctgtgagcctgcatgggatggatgacccggagagagaa
gtgttagagtggaggtttgacagccgcctagcatttcatcacgtggcccgagagctgcatccggagtacttcaagaactgctgatatcgagcttgctacaagggactttccgctggggactttcca
gggaggcgtggcctgggcgggactggggagtggcgagccctcagatcctgcatataagcagctgctttttgcctgtactgggtctctctggttagaccagatctgagcctgggagctctctggct
aactagggaacccactgcttaagcctcaataaagcttgccttgagtgcttcaagtagtgtgtgcccgtctgttgtgtgactctggtaactagagatccctcagacccttttagtcagtgtggaaaatct
ctagcagtggcgcccgaacagggacttgaaagcgaaagggaaaccagaggagctctctcgacgcaggactcggcttgctgaagcgcgcacggcaagaggcgaggggcggcgactggtga
gtacgccaaaaattttgactagcggaggctagaaggagagagatgggtgcgagagcgtcagtattaagcgggggagaattagatcgcgatgggaaaaaattcggttaaggccagggggaa
agaaaaaatataaattaaaacatatagtatgggcaagcagggagctagaacgattcgcagttaatcctggcctgttagaaacatcagaaggctgtagacaaatactgggacagctacaaccatc
ccttcagacaggatcagaagaacttagatcattatataatacagtagcaaccctctattgtgtgcatcaaaggatagagataaaagacaccaaggaagctttagacaagatagaggaagagcaa
aacaaaagtaagaccaccgcacagcaagcggccggccgctgatcttcagacctggaggaggagatatgagggacaattggagaagtgaattatataaatataaagtagtaaaaattgaacca
ttaggagtagcacccaccaaggcaaagagaagagtggtgcagagagaaaaaagagcagtgggaataggagctttgttccttgggttcttgggagcagcaggaagcactatgggcgcagcgt
caatgacgctgacggtacaggccagacaattattgtctggtatagtgcagcagcagaacaatttgctgagggctattgaggcgcaacagcatctgttgcaactcacagtctggggcatcaagca
gctccaggcaagaatcctggctgtggaaagatacctaaaggatcaacagctcctggggatttggggttgctctggaaaactcatttgcaccactgctgtgccttggaatgctagttggagtaataa
atctctggaacagatttggaatcacacgacctggatggagtgggacagagaaattaacaattacacaagcttaatacactccttaattgaagaatcgcaaaaccagcaagaaaagaatgaacaa
gaattattggaattagataaatgggcaagtttgtggaattggtttaacataacaaattggctgtggtatataaaattattcataatgatagtaggaggcttggtaggtttaagaatagtttttgctgta
ctttctatagtgaatagagttaggcagggatattcaccattatcgtttcagacccacctcccaaccccgaggggacccgacaggcccgaaggaatagaagaagaaggtggagagagagacag
agacagatccattcgattagtgaacggatctcgacggtatcgcctttaaaagaaaaggggggattggggggtacagtgcaggggaaagaatagtagacataatagcaacagacatacaaact
aaagaattacaaaaacaaattacaaaaattcaaaattttcgggtttattacagggacagcagagatccagtttatcgataagcttgggagttccgcgttacataacttacggtaaatggcccgcctg
gctgaccgcccaacgacccccgcccattgacgtcaataatgacgtatgttcccatagtaacgccaatagggactttccattgacgtcaatgggtggagtatttacggtaaactgcccacttggcagt
acatcaagtgtatcatatgccaagtacgccccctattgacgtcaatgacggtaaatggcccgcctggcattatgcccagtacatgaccttatgggactttcctacttggcagtacatctacgtattagt
catcgctattaccatggtgatgcggttttggcagtacatcaatgggcgtggatagcggtttgactcacggggatttccaagtctccaccccattgacgtcaatgggagtttgttttggcaccaaaatc
aacgggactttccaaaatgtcgtaacaactccgccccattgacgcaaatgggcggtaggcgtgtacggtgggaggtctatataagcagagctcgtttagtgaaccgtcagatcgcctggagacg
ccatccacgctgttttgacctccatagaagacaccgactctactagaggatctaccggtcgccaccatggccctgtccaacaagttcatcggcgacgacatgaagatgacctaccacatggacggc
tgcgtgaacggccactacttcaccgtgaagggcgagggcagcggcaagccctacgagggcacccagacctccaccttcaaggtgaccatggccaacggcggccccctggccttctccttcgac

atcctgtccaccgtgttcatgtacggcaaccgctgcttcaccgcctaccccaccagcatgcccgactacttcaagcaggccttccccgacggcatgtcctacgagagaaccttcacctacgaggacg
gcggcgtggccaccgccagctgggagatcagcctgaagggcaactgcttcgagcacaagtccaccttccacggcgtgaacttccccgccgacggccccgtgatggccaagaagaccaccggc
tgggacccctccttcgagaagatgaccgtgtgcgacggcatcttgaagggcgacgtgaccgccttcctgatgctgcagggcggcggcaactacagatgccagttccacacctcctacaagacca
agaagcccgtgaccatgcccccaaccacgtggtggagcaccgcatcgccagaaccgacctggacaagggcggcaacagcgtgcagctgaccgagcacgccgtggcccacatcacctccgt
ggtgcccttctccggactcagatctcgagctcaagcttcgaattctgcagtcgacggtaccgcgggcccgggatccaccggatctagataactgatcataattctaccgggtaggggaggcgcttt
tcccaaggcagtctggagcatgcgctttagcagccccgctgggcacttggcgctacacaagtggcctctggcctcgcacacattccacatccaccggtaggcgccaaccggctccgttctttggtg
gccccttcgcgccaccttctactcctcccctagtcaggaagttcccccccgccccgcagctcgcgtcgtgcaggacgtgacaaatggaagtagcacgtctcactagtctcgtgcagatggacagca
ccgctgagcaatggaagcgggtaggcctttggggcagcggccaatagcagctttgctccttcgctttctgggctcagaggctgggaaggggtgggtccgggggcgggctcaggggcgggct
caggggcggggcgggcgcccgaaggtcctccggaggcccggcattctgcacgcttcaaaagcgcacgtctgccgcgctgttctcctcttcctcatctccgggcctttcgacctgcagcccaagctt
accatgaccgagtacaagcccacggtgcgcctcgccacccgcgacgacgtccccagggccgtacgcaccctcgccgccgcgttcgccgactaccccgccacgcgccacaccgtcgatccggac
cgccacatcgagcgggtcaccgagctgcaagaactcttcctcacgcgcgtcgggctcgacatcggcaaggtgtgggtcgcggacgacggcgccgcggtggcggtctggaccacgccggaga
gcgtcgaagcgggggcggtgttcgccgagatcggcccgcgcatggccgagttgagcggttcccggctggccgcgcagcaacagatggaaggcctcctggcgccgcaccggcccaaggagc
ccgcgtggttcctggccaccgtcggcgtctcgcccgaccaccagggcaagggtctgggcagcgccgtcgtgctccccggagtggaggcggccgagcgcgccggggtgcccgccttcctgga
gacctccgcgccccgcaacctccccttctacgagcggctcggcttcaccgtcaccgccgacgtcgaggtgcccgaaggaccgcgcacctggtgcatgacccgcaagcccggtgcctgaccgcgt
ctggaacaatcaacctctggattacaaaatttgtgaaagattgactggtattcttaactatgttgctccttttacgctatgtggatacgctgctttaatgcctttgtatcatgctattgcttcccgtatggctt
tcattttctcctccttgtataaatcctggttgctgtctctttatgaggagttgtggcccgttgtcaggcaacgtggcgtggtgtgcactgtgtttgctgacgcaacccccactggttggggcattgccacc
acctgtcagctcctttccgggactttcgctttccccctccctattgccacggcggaactcatcgccgcctgccttgcccgctgctggacaggggctcggctgttgggcactgacaattccgtggtgttg
tcggggaagctgacgtcctttccatggctgctcgcctgtgttgccacctggattctgcgcgggacgtccttctgctacgtcccttcggccctcaatccagcggaccttccttcccgcggcctgctgccg
gctctgcggcctcttccgcgtcttcgccttcgccctcagacgagtcggatctccctttgggccgcctccccgcctggaattaattctgcagtcgagacctagaaaaacatggagcaatcacaagtag
caatacagcagctaccaatgctgattgtgcctggctagaagcacaagaggaggaggaggtgggttttccagtcacacctcaggtacctttaagaccaatgacttacaaggcagctgtagatctta
gccactttttaaaagaaaagaggggactggaagggctaattcactcccaacgaagacaagatatccttgatctgtggatctaccacacacaaggctacttccctgattagcagaactacacaccag
ggccaggggtcagatatccactgacctttggatggtgctacaagctagtaccagttgagccagataaggtagaagaggccaataaaggagagaacaccagcttgttacaccctgtgagcctgc
atgggatggatgacccggagagagaagtgttagagtggaggtttgacagccgcctagcatttcatcacgtggcccgagagctgcatccggagtacttcaagaactgctgatatcgagcttgcta
caagggactttccgctggggactttccagggaggcgtggcctgggcgggactggggagtggcgagccctcagatcctgcatataagcagctgctttttgcctgtactgggtctctctggttagac
cagatctgagcctgggagctctctggctaactagggaacccactgcttaagcctcaataaagcttgccttgagtgcttcaagtagtgtgtgcccgtctgttgtgtgactctggtaactagagatccct
cagacccttttagtcagtgtggaaaatctctagcagtagtagttcatgtcatcttattattcagtatttataacttgcaaagaaatgaatatcagagagtgagaggccttgacattgctagcgttttacc
gtcgacctctagctagagcttggcgtaatcatggtcatagctgtttcctgtgtgaaattgttatccgctcacaattccacacaacatacgagccggaagcataaagtgtaaagcctggggtgcctaat
gagtgagctaactcacattaattgcgttgcgctcactgcccgctttccagtcgggaaacctgtcgtgccagctgcattaatgaatcggccaacgcgcggggagaggcggtttgcgtattgggcgct
cttccgcttcctcgctcactgactcgctgcgctcggtcgttcggctgcggcgagcggtatcagctcactcaaaggcggtaatacggttatccacagaatcaggggataacgcaggaaagaacatg
tgagcaaaaggccagcaaaaggccaggaaccgtaaaaaggccgcgttgctggcgtttttccataggctccgcccccctgacgagcatcacaaaaatcgacgctcaagtcagaggtggcgaaa
cccgacaggactataaagataccaggcgtttccccctggaagctccctcgtgcgctctcctgttccgaccctgccgcttaccggatacctgtccgcctttctcccttcgggaagcgtggcgctttctcat
agctcacgctgtaggtatctcagttcggtgtaggtcgttcgctccaagctgggctgtgtgcacgaaccccccgttcagcccgaccgctgcgccttatccggtaactatcgtcttgagtccaacccggt
aagacacgacttatcgccactggcagcagccactggtaacaggattagcagagcgaggtatgtaggcggtgctacagagttcttgaagtggtggcctaactacggctacactagaagaacagt
atttggtatctgcgctctgctgaagccagttaccttcggaaaaagagttggtagctcttgatccggcaaacaaaccaccgctggtagcggtggtttttttgtttgcaagcagcagattacgcgcaga
aaaaaaggatctcaagaagatcctttgatcttttctacggggtctgacgctcagtggaacgaaaactcacgttaagggattttggtcatgagattatcaaaaaggatcttcacctagatccttttaaat
taaaaatgaagttttaaatcaatctaaagtatatatgagtaaacttggtctgacagttaccaatgcttaatcagtgaggcacctatctcagcgatctgtctatttcgttcatccatagttgcctgactcccc
gtcgtgtagataactacgatacgggagggcttaccatctggccccagtgctgcaatgataccgcgagacccacgctcaccggctccagatttatcagcaataaaccagccagccggaagggccg
agcgcagaagtggtcctgcaactttatccgcctccatccagtctattaattgttgccgggaagctagagtaagtagttcgccagttaatagtttgcgcaacgttgttgccattgctacaggcatcgtg
gtgtcacgctcgtcgtttggtatggcttcattcagctccggttcccaacgatcaaggcgagttacatgatcccccatgttgtgcaaaaaagcggttagctccttcggtcctccgatcgttgtcagaagt
aagttggccgcagtgttatcactcatggttatggcagcactgcataattctcttactgtcatgccatccgtaagatgcttttctgtgactggtgagtactcaaccaagtcattctgagaatagtgtatgc
ggcgaccgagttgctcttgcccggcgtcaatacgggataataccgcgccacatagcagaactttaaaagtgctcatcattggaaaacgttcttcggggcgaaaactctcaaggatcttaccgctgt
tgagatccagttcgatgtaacccactcgtgcacccaactgatcttcagcatcttttactttcaccagcgtttctgggtgagcaaaaacaggaaggcaaaatgccgcaaaaaagggaataagggcg
acacggaaatgttgaatactcatactcttcctttttcaatattattgaagcatttatcagggttattgtctcatgagcggatacatatttgaatgtatttagaaaaataaacaaataggggttccgcgcac
atttccccgaaaagtgccacctgacgtcgacggatcgggagatcaacttgtttattgcagcttataatggttacaaataaagcaatagcatcacaaatttcacaaataaagcatttttttcactgcattc
tagttgtggtttgtccaaactcatcaatgtatcttatcatgtctggatcaactggataactcaagctaaccaaaatcatcccaaacttcccaccccataccctattaccactgccaattacctgtggtttcat
ttactctaaacctgtgattcctctgaattattttcattttaaagaaattgtatttgttaaatatgtactacaaacttagtagt

Functional DNA region	Line number	Base number
5′ LTR	L1-L6	1-635
PBS (primer binding site)	L6	636-653
Ψ (*\ packaging signal)	L7	685-822
RRE (Rev-response element)	L11-L13	1303-536
cPTT/CTS (central polypurine tract/central termination sequence)	L17-L18	2028-2151

PCMV IE (human cytomegalovirus immediate early promoter)	L18-L23	2185-2787
Reverse-1 primer binding site	L23	2787-2806
AmCyan1 fluorescent protein gene	L23-L29	2807-3493
Forward-1 primer binding site	L28	3426-3445
MCS (Multiple cloning site)	L29	3507-3559
EcoRI restriction site (3523-3528)	L29	3523-3528
BamHI restriction site	L29	3554-3559
STOP codons	L29	3568-3578
PPGK (phosphoglycerate kinase promoter)	L29-L33	3583-4091
Puro^R (Puromycin resistance gene)	L34-L38	4112-4711
WPRE (woodchuck posttranscriptional regulatory element)	L39-L43	4725-5316
3′ LTR	L45-L50	5519-6155
pUC origin of replication (complementary)	L53-L59	6625-7298
Amp^R (Ampicillin resistance gene; β-lactamase)(complementary)	L60-L68	7443-8439

Table S3. DNA sequence of pE2-Crimson prokaryotic expression plasmid. Functional regions are highlighted with different colours. Note the binding sites of Forward-2 (Line-3) and Reverse-2 (Line-8) primers.

agcgcccaatacgcaaaccgcctctccccgcgcgttggccgattcattaatgcagctggcacgacaggtttcccgactggaaagcgggcagtgagcgcaacgcaattaatgtgagttagctcact
cattaggcaccccaggctttacactttatgcttccggctcgtatgttgtgtggaattgtgagcggataacaatttcacacaggaaacagctatgaccatgattacgccaagcttgcatgcctgcaggt
cgactctagaggatccccgggtaccggtcgccaccatggatagcactgagaacgtcatcaagcccttcatgcgcttcaaggtgcacatggagggctccgtgaacggccacgagttcgagatcga
gggcgtgggcgagggcaagccctacgagggcacccagaccgccaagctgcaagtgaccaagggcggcccctgcccttcgcctgggacatcctgtccccccagttcttctacggctccaaggc
gtacatcaagcaccccgccgacatccccgactacctcaagcagtccttccccgagggcttcaagtgggagcgcgtgatgaacttcgaggacggcggcgtggtgaccgtgacccaggactcctcc
ctgcaggacggcaccctcatctaccacgtgaagttcatcggcgtgaacttcccctccgacggccccgtaatgcagaagaagactctgggctgggagccctccactgagcgcaactaccccgcg
acggcgtgctgaagggcgagaaccacatggcgctgaagctgaagggcggcggccactacctgtgtgagttcaagtccatctacatggccaagaagcccgtgaagctgcccggctaccactac
gtggactacaagctcgacatcacctcccacaacgaggactacaccgtggtggagcagtacgagcgcgccgaggcccgccaccacctgttccagtagcggccgcgactctagaattccaactga
gcgccggtcgctaccattaccaacttgtctggtgtcaaaaataataggcctactagtcggccgtacgggccctttcgtctcgcgcgtttcggtgatgacggtgaaaacctctgacacatgcagctcc
cggagacggtcacagcttgtctgtaagcggatgccgggagcagacaagcccgtcagggcgcgtcagcgggtgttggcgggtgtcggggctggcttaactatgcggcatcagagcagattgta
ctgagagtgcaccatatgcggtgtgaaataccgcacagatgcgtaaggagaaaataccgcatcaggcggccttaagggcctcgtgatacgcctatttttataggttaatgtcatgataataatggt
ttcttagacgtcaggtggcacttttcggggaaatgtgcgcggaacccctatttgtttatttttctaaatacattcaaatatgtatccgctcatgagacaataaccctgataaatgcttcaataatattgaa
aaaggaagagtatgagtattcaacatttccgtgtcgcccttattcccttttttgcggcattttgccttcctgtttttgctcacccagaaacgctggtgaaagtaaaagatgctgaagatcagttgggtgc
acgagtgggttacatcgaactggatctcaacagcggtaagatccttgagagttttcgccccgaagaacgttttccaatgatgagcacttttaaagttctgctatgtggcgcggtattatcccgtattg
acgccgggcaagagcaactcggtcgccgcatacactattctcagaatgacttggttgagtactcaccagtcacagaaaagcatcttacggatggcatgacagtaagagaattatgcagtgctgcc
ataaccatgagtgataacactgcggccaacttacttctgacaacgatcggaggaccgaaggagctaaccgcttttttgcacaacatgggggatcatgtaactcgccttgatcgttgggaaccgga
gctgaatgaagccataccaaacgacgagcgtgacaccacgatgcctgtagcaatggcaacaacgttgcgcaaactattaactggcgaactacttactctagcttcccggcaacaattaatagact
ggatggaggcggataaagttgcaggaccacttctgcgctcggcccttccggctggctggtttattgctgataaatctggagccggtgagcgtgggtctcgcggtatcattgcagcactggggcca
gatggtaagccctcccgtatcgtagttatctacacgacggggagtcaggcaactatggatgaacgaaatagacagatcgctgagataggtgcctcactgattaagcattggtaactgtcagacca
agtttactcatatatactttagattgatttaaaacttcatttttaatttaaaaggatctaggtgaagatcctttttgataatctcatgaccaaaatcccttaacgtgagttttcgttccactgagcgtcagacc
ccgtagaaaagatcaaaggatcttcttgagatcctttttttctgcgcgtaatctgctgcttgcaaacaaaaaaaccaccgctaccagcggtggtttgtttgccggatcaagagctaccaactctttttcc
gaaggtaactggcttcagcagagcgcagataccaaatactgttcttctagtgtagccgtagttaggccaccacttcaagaactctgtagcaccgcctacatacctcgctctgctaatcctgttaccag
tggctgctgccagtggcgataagtcgtgtcttaccgggttggactcaagacgatagttaccggataaggcgcagcggtcgggctgaacggggggttcgtgcacacagcccagcttggagcga
acgacctacaccgaactgagatacctacagcgtgagctatgagaaagcgccacgcttcccgaagggagaaaggcggacaggtatccggtaagcggcagggtcggaacaggagagcgcac
gagggagcttccagggggaaacgcctggtatctttatagtcctgtcgggtttcgccacctctgacttgagcgtcgatttttgtgatgctcgtcaggggggcggagcctatggaaaaacgccagca
acgcggcctttttacggttcctggccttttgctggccttttgctcacatgttctttcctgcgttatcccctgattctgtggataaccgtattaccgcctttgagtgagctgataccgctcgccgcagccgaa
cgaccgagcgcagcgagtcagtgagcgaggaagcggaag

Functional DNA region	Line number	Base number
Plac (lac promoter)	L1-L2	95-178
Ribosome binding site	L2	206-209
Start codon	L2	217-219
5′ MCS (multiple cloning site)	L2-L3	234-281
Forward-2 primer binding site	L3	289-309
E2-Crimson fluorescent protein gene	L3-L8	289-966
Reverse-2 primer binding site	L8	946-966
3′ MCS (multiple cloning site)	L8-L9	966-1065
Amp^R (Ampicillin resistance gene; β-lactamase)	L13-L19	1511-2371
pUC origin of replication	L21-L26	2519-3161

Table S4. DNA sequence of pZsYellow prokaryotic expression plasmid. Functional regions are highlighted with different colours. Note the binding sites of Forward-3 (Line-3) and Reverse-3 (Line-8) primers.

agcgcccaatacgcaaaccgcctctccccgcgcgttggccgattcattaatgcagctggcacgacaggtttcccgactggaaagcgggcagtgagcgcaacgcaattaatgtgagttagctcact
cattaggcaccccaggctttacactttatgcttccggctcgtatgttgtgtggaattgtgagcggataacaatttcacacaggaaacagctatgaccatgattacgccaagcttgcatgcctgcaggt
cgactctagaggatccccgggtaccggtcgccaccatggctcattcaaagcacggtctaaaagaagaaatgacaatgaaataccacatggaagggtgcgtcaacggacataaatttgtgatcac
gggcgaaggcattggatatccgttcaaagggaaacagactattaatctgtgtgtgatcgaaggggggaccattgccattttccgaagacatattgtcagctggctttaagtacggagacaggatttt
cactgaatatcctcaagacatagtagactatttcaagaactcgtgtcctgctggatatacatggggcaggtcttttctctttgaggatggagcagtctgcatatgcaatgtagatataacagtgagtg
tcaaagaaaactgcatttatcataagagcatatttaatggagtgaattttcctgctgatggacctgtgatgaaaaagatgacaactaactgggaagcatcctgcgagaagatcatgccagtaccta
agcaggggatactgaaaggggatgtctccatgtacctccttctgaaggatggtgggcgttaccggtgccagttcgacacagtttacaaagcaaagtctgtgccaagtaagatgccggagtggca
cttcatccagcataagctcctccgtgaagaccgcagcgatgctaagaatcagaagtggcagctgacagagcatgctattgcattcccttctgccttggcctgaagcggccgcgactctagaattcc
aactgagcgccggtcgctaccattaccaacttgtctggtgtcaaaaataataggcctactagtcggccgtacgggccctttcgtctcgcgcgtttcggtgatgacggtgaaaacctctgacacatgc
agctcccggagacggtcacagcttgtctgtaagcggatgccgggagcagacaagcccgtcagggcgcgtcagcgggtgttggcgggtgtcggggctggcttaactatgcggcatcagagca
gattgtactgagagtgcaccatatgcggtgtgaaataccgcacagatgcgtaaggagaaaataccgcatcaggcggccttaagggcctcgtgatacgcctatttttataggttaatgtcatgataa
taatggtttcttagacgtcaggtggcacttttcggggaaatgtgcgcggaacccctatttgtttatttttctaaatacattcaaatatgtatccgctcatgagacaataaccctgataaatgcttcaataat
attgaaaaaggaagagtatgagtattcaacatttccgtgtcgcccttattcccttttttgcggcattttgccttcctgtttttgctcacccagaaacgctggtgaaagtaaaagatgctgaagatcagttg
ggtgcacgagtgggttacatcgaactggatctcaacagcggtaagatccttgagagttttcgccccgaagaacgttttccaatgatgagcacttttaaagttctgctatgtggcgcggtattatcccg
tattgacgccgggcaagagcaactcggtcgccgcatacactattctcagaatgacttggttgagtactcaccagtcacagaaaagcatcttacggatggcatgacagtaagagaattatgcagtg
ctgccataaccatgagtgataacactgcggccaacttacttctgacaacgatcggaggaccgaaggagctaaccgcttttttgcacaacatgggggatcatgtaactcgccttgatcgttgggaac
cggagctgaatgaagccataccaaacgacgagcgtgacaccacgatgcctgtagcaatggcaacaacgttgcgcaaactattaactggcgaactacttactctagcttcccggcaacaattaata
gactggatggaggcggataaagttgcaggaccacttctgcgctcggcccttccggctggctggtttattgctgataaatctggagccggtgagcgtgggtctcgcggtatcattgcagcactggg
gccagatggtaagccctcccgtatcgtagttatctacacgacggggagtcaggcaactatggatgaacgaaatagacagatcgctgagataggtgcctcactgattaagcattggtaactgtcag
accaagtttactcatatatactttagattgatttaaaacttcatttttaatttaaaaggatctaggtgaagatcctttttgataatctcatgaccaaaatcccttaacgtgagttttcgttccactgagcgtca
gaccccgtagaaaagatcaaaggatcttcttgagatcctttttttctgcgcgtaatctgctgcttgcaaacaaaaaaaccaccgctaccagcggtggtttgtttgccggatcaagagctaccaactctt
tttccgaaggtaactggcttcagcagagcgcagataccaaatactgttcttctagtgtagccgtagttaggccaccacttcaagaactctgtagcaccgcctacatacctcgctctgctaatcctgtta
ccagtggctgctgccagtggcgataagtcgtgtcttaccgggttggactcaagacgatagttaccggataaggcgcagcggtcgggctgaacggggggttcgtgcacacagcccagcttgga
gcgaacgacctacaccgaactgagatacctacagcgtgagctatgagaaagcgccacgcttcccgaagggagaaaggcggacaggtatccggtaagcggcagggtcggaacaggagagc
gcacgagggagcttccagggggaaacgcctggtatctttatagtcctgtcgggtttcgccacctctgacttgagcgtcgatttttgtgatgctcgtcaggggggcggagcctatggaaaaacgcc
agcaacgcggcctttttacggttcctggccttttgctggccttttgctcacatgttctttcctgcgttatcccctgattctgtggataaccgtattaccgcctttgagtgagctgataccgctcgccgcagc
cgaacgaccgagcgcagcgagtcagtgagcgaggaagcggaag

Functional DNA region	Line number	Base number
P_{lac} (lac promoter)	L1-L2	95-178
Ribosome binding site	L2	206-209
Start codon	L2	217-219
5′ MCS (multiple cloning site)	L2-L3	234-292
Forward-3 primer binding site	L3	289-309
ZsYellow fluorescent protein gene	L3-L8	289-984
Reverse-3 primer binding site	L8	964-984
3′ MCS (multiple cloning site)	L8-L9	986-1085
Amp^R (Ampicillin resistance gene; β-lactamase)	L13-L19	1531-2391
pUC origin of replication	L21-L26	2539-3181

Table S5. DNA alignment of the theorised (Query) and engineered (Sbjct) sequences of pLVX-E2.Crimson-C1 plasmid. A forward primer (5′ gtacggtgggaggtctatat 3′) was used for the sequencing.

```
Query  2721  CGTCAGATCGCCTGGAGACGCCATCCACGCTGTTTTGACCTCCATAGAAGACACCGACTC  2780
             ||||||||||||||||||||||||||||||||||||||||||||||||||||||||||||
Sbjct  9     CGTCAGATCGCCTGGAGACGCCATCCACGCTGTTTTGACCTCCATAGAAGACACCGACTC  68

Query  2781  TACTAGAGGATCTACCGGTCGCCACCCTCGAGATGGATAGCACTGAGAACGTCATCAAGC  2840
             ||||||||||||||||||||||||||||||||||||||||||||||||||||||||||||
Sbjct  69    TACTAGAGGATCTACCGGTCGCCACCCTCGAGATGGATAGCACTGAGAACGTCATCAAGC  128

Query  2841  CCTTCATGCGCTTCAAGGTGCACATGGAGGGCTCCGTGAACGGCCACGAGTTCGAGATCG  2900
             ||||||||||||||||||||||||||||||||||||||||||||||||||||||||||||
Sbjct  129   CCTTCATGCGCTTCAAGGTGCACATGGAGGGCTCCGTGAACGGCCACGAGTTCGAGATCG  188

Query  2901  AGGGCGTGGGCGAGGGCAAGCCCTACGAGGGCACCCAGACCGCCAAGCTGCAAGTGACCA  2960
             ||||||||||||||||||||||||||||||||||||||||||||||||||||||||||||
Sbjct  189   AGGGCGTGGGCGAGGGCAAGCCCTACGAGGGCACCCAGACCGCCAAGCTGCAAGTGACCA  248

Query  2961  AGGGCGGCCCCCTGCCCTTCGCCTGGGACATCCTGTCCCCCCAGTTCTTCTACGGCTCCA  3020
             ||||||||||||||||||||||||||||||||||||||||||||||||||||||||||||
Sbjct  249   AGGGCGGCCCCCTGCCCTTCGCCTGGGACATCCTGTCCCCCCAGTTCTTCTACGGCTCCA  308

Query  3021  AGGCGTACATCAAGCACCCCGCCGACATCCCCGACTACCTCAAGCAGTCCTTCCCCGAGG  3080
             ||||||||||||||||||||||||||||||||||||||||||||||||||||||||||||
Sbjct  309   AGGCGTACATCAAGCACCCCGCCGACATCCCCGACTACCTCAAGCAGTCCTTCCCCGAGG  368

Query  3081  GCTTCAAGTGGGAGCGCGTGATGAACTTCGAGGACGGCGGCGTGGTGACCGTGACCCAGG  3140
             ||||||||||||||||||||||||||||||||||||||||||||||||||||||||||||
Sbjct  369   GCTTCAAGTGGGAGCGCGTGATGAACTTCGAGGACGGCGGCGTGGTGACCGTGACCCAGG  428

Query  3141  ACTCCTCCCTGCAGGACGGCACCCTCATCTACCACGTGAAGTTCATCGGCGTGAACTTCC  3200
             ||||||||||||||||||||||||||||||||||||||||||||||||||||||||||||
Sbjct  429   ACTCCTCCCTGCAGGACGGCACCCTCATCTACCACGTGAAGTTCATCGGCGTGAACTTCC  488

Query  3201  CCTCCGACGGCCCCGTAATGCAGAAGAAGACTCTGGGCTGGGAGCCCTCCACTGAGCGCA  3260
             ||||||||||||||||||||||||||||||||||||||||||||||||||||||||||||
Sbjct  489   CCTCCGACGGCCCCGTAATGCAGAAGAAGACTCTGGGCTGGGAGCCCTCCACTGAGCGCA  548

Query  3261  ACTACCCCCGCGACGGCGTGCTGAAGGGCGAGAACCACATGGCGCTGAAGCTGAAGGGCG  3320
             ||||||||||||||||||||||||||||||||||||||||||||||||||||||||||||
Sbjct  549   ACTACCCCCGCGACGGCGTGCTGAAGGGCGAGAACCACATGGCGCTGAAGCTGAAGGGCG  608

Query  3321  GCGGCCACTACCTGTGTGAGTTCAAGTCCATCTACATGGCCAAGAAGCCCGTGAAGCTGC  3380
             ||||||||||||||||||||||||||||||||||||||||||||||||||||||||||||
Sbjct  609   GCGGCCACTACCTGTGTGAGTTCAAGTCCATCTACATGGCCAAGAAGCCCGTGAAGCTGC  668

Query  3381  CCGGCTACCACTACGTGGACTACAAGCTCGACATCACCTCCCACAACGAGGACTACACCG  3440
             ||||||||||||||||||||||||||||||||||||||||||||||||||||||||||||
Sbjct  669   CCGGCTACCACTACGTGGACTACAAGCTCGACATCACCTCCCACAACGAGGACTACACCG  728

Query  3441  TGGTGGAGCAGTACGAGCGCCGAGGCCCGCCACCACCTGTTCCAGTAGGAATTCTG-C  3499
             ||||||||||||||||||||||||||||||||||||||||| |||||||||||||| |
Sbjct  729   TGGTGGAGCAGTACGAGCGCCGAGGCCCGCCACCACCTGTTTCAGTAGGAATTCTGCC  788

Query  3500  AGTCGACGGTACCGCGGGCCCGGGATCCACCGGA-TCTAGATAACTGATCATAATTCTAC  3558
             ||||||||||||||||| |||||||||||||||| ||||||||||||||||||||| || 
Sbjct  789   AGTCGACGGTACCGCGGCCCCGGGATCCACCGGATTCTAGATAACTGATCATAATT-TAG  847

Query  3559  CGGGTAGGGGAGGCGCTTTTCCC-A-AGGCAGTCTGGAGCAT-GCGCTTTAGCAGCCCCG  3615
             |||| |||||||||||||||||| | |||||||||||||| | |  |||   || |||| 
Sbjct  848   CGGGAAGGGGAGGCGCTTTTCCCCAGAGGCAGTCTGGAGCGTTGTACTTACCCACCCCCT  907

Query  3616  CTGGGCACTTGGCG  3629
             | |||||| |||||
Sbjct  908   CGGGGCACCTGGCG  921
```

Functional DNA region	Base number (on Query seq.)
pLVX backbone (flanking 5′ end of E2.Crimson gene)	← 2721-2806
XhoI restriction site	2807-2812
Start codon	2813-2815
E2.Crimson fluorescent protein gene	2813-3490
Stop codon	3488-3490
EcoRI restriction site	3491-3496
pLVX backbone (flanking 3′ end of E.2 Crimson gene)	3497-3629 →
Triple Stop codons (located in the backbone of pLVX plasmid)	3536-3546

Table S6. DNA alignment of the theorised (Query) and engineered (Sbjct) sequences of pLVX-E2.Crimson-C1 plasmid. A reverse primer (5′ cagactgccttgggaaaa 3′) was used for the sequencing.

```
Query  2944  CAAGCTGCAAGTGACCAAGGGCGGCCCCCTGCCCTTCGCCTGGG-ACATCCTGTCCCCCC  3002
             |||||||| |||||||||| |||||||||||||||||||||||| |||||||||||||||
Sbjct  613   CAAGCTGCTAGTGACCAAGTGCGGCCCCCTGCCCTTCGCCTGGGTACATCCTGTCCCCCC  554

Query  3003  AGTTCTTCTACGGCTCCAAGGCGTACATCAAGCACCCCGCCGACATCCCCGACTACCTCA  3062
             |||| |||||||||||||||||||||||||||||||||||||||||||||||||||||||
Sbjct  553   AGTT-TTCTACGGCTCCAAGGCGTACATCAAGCACCCCGCCGACATCCCCGACTACCTCA  495

Query  3063  AGCAGTCCTTCCCCGAGGGCTTCAAGTGGGAGCGCGTGATGAACTTCGAGGACGGCGGCG  3122
             ||||||||||||||||||||||||||||||||||||||||||||||||||||||||||||
Sbjct  494   AGCAGTCCTTCCCCGAGGGCTTCAAGTGGGAGCGCGTGATGAACTTCGAGGACGGCGGCG  435

Query  3123  TGGTGACCGTGACCCAGGACTCCTCCCTGCAGGACGGCACCCTCATCTACCACGTGAAGT  3182
             ||||||||||||||||||||||||||||||||||||||||||||||||||||||||||||
Sbjct  434   TGGTGACCGTGACCCAGGACTCCTCCCTGCAGGACGGCACCCTCATCTACCACGTGAAGT  375

Query  3183  TCATCGGCGTGAACTTCCCCTCCGACGGCCCCGTAATGCAGAAGAAGACTCTGGGCTGGG  3242
             ||||||||||||||||||||||||||||||||||||||||||||||||||||||||||||
Sbjct  374   TCATCGGCGTGAACTTCCCCTCCGACGGCCCCGTAATGCAGAAGAAGACTCTGGGCTGGG  315

Query  3243  AGCCCTCCACTGAGCGCAACTACCCCCGCGACGGCGTGCTGAAGGGCGAGAACCACATGG  3302
             ||||||||||||||||||||||||||||||||||||||||||||||||||||||||||||
Sbjct  314   AGCCCTCCACTGAGCGCAACTACCCCCGCGACGGCGTGCTGAAGGGCGAGAACCACATGG  255

Query  3303  CGCTGAAGCTGAAGGGCGGCGGCCACTACCTGTGTGAGTTCAAGTCCATCTACATGGCCA  3362
             ||||||||||||||||||||||||||||||||||||||||||||||||||||||||||||
Sbjct  254   CGCTGAAGCTGAAGGGCGGCGGCCACTACCTGTGTGAGTTCAAGTCCATCTACATGGCCA  195

Query  3363  AGAAGCCCGTGAAGCTGCCCGGCTACCACTACGTGGACTACAAGCTCGACATCACCTCCC  3422
             ||||||||||||||||||||||||||||||||||||||||||||||||||||||||||||
Sbjct  194   AGAAGCCCGTGAAGCTGCCCGGCTACCACTACGTGGACTACAAGCTCGACATCACCTCCC  135

Query  3423  ACAACGAGGACTACACCGTGGTGGAGCAGTACGAGCGCGCCGAGGCCCGCCACCACCTGT  3482
             ||||||||||||||||||||||||||||||||||||||||||||||||||||||||||||
Sbjct  134   ACAACGAGGACTACACCGTGGTGGAGCAGTACGAGCGCGCCGAGGCCCGCCACCACCTGT  75

Query  3483  TCCAGTAGGAATTCTGCAGTCGACGGTACCGCGGGCCCGGGATCCACCGGATCTAGATAA  3542
             ||||||||||||||||||||||||||||||||||||||||||||||||||||||||||||
Sbjct  74    TCCAGTAGGAATTCTGCAGTCGACGGTACCGCGGGCCCGGGATCCACCGGATCTAGATAA  15

Query  3543  CTGATCATA  3551
             |||||||||
Sbjct  14    CTGATCATA  6
```

Functional DNA region	Base number (on Query seq.)
E2.Crimson fluorescent protein gene (covering 132-678 bases)	← 2944-3490
Stop codon	3488-3490
EcoRI restriction site	3491-3496
pLVX backbone (flanking 3′ end of E.2 Crimson gene)	3497-3551→
Triple Stop codons (located in the backbone of pLVX plasmid)	3536-3546

Table S7. Primary glioblastoma cell lines that were successfully infected with lentiviral particles. The protocol in Results Section 3.6 "Preparation and purification of lentiviral particles" was used to prepare the lentiviral particles.

	Cell line [1,2]	Reference no.		Cell line	Reference no.		Cell line	Reference no.
**1**	BAH1	QIMR-B001	**5**	MMK1	QIMR-B001	**9**	RN1	QIMR-B001
**2**	FPW1	QIMR-B001	**6**	MN1	QIMR-B001	**10**	SB2b	QIMR-B001
**3**	HW1	QIMR-B001	**7**	PB1	QIMR-B001	**11**	SJH1	QIMR-B001
**4**	JK2	QIMR-B001	**8**	RKI1	QIMR-B001	**12**	WK1	QIMR-B001

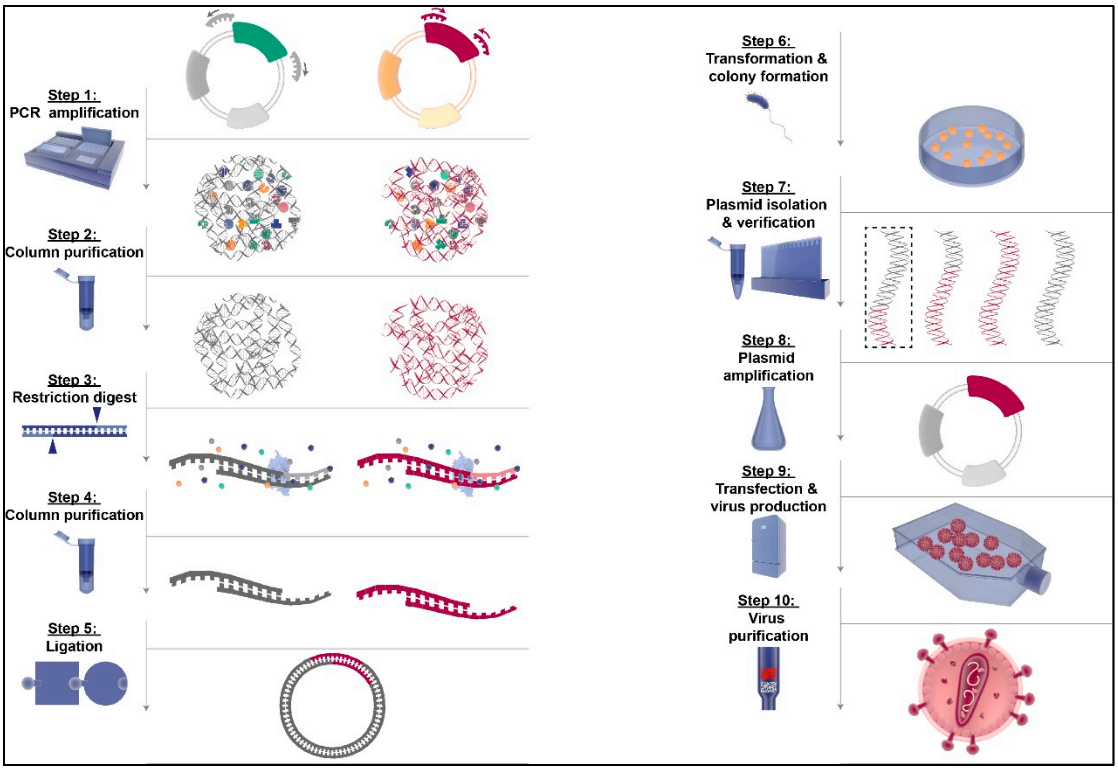

Figure S1. Outline of High-Fidelity Amplification-Restriction-Ligation strategy.

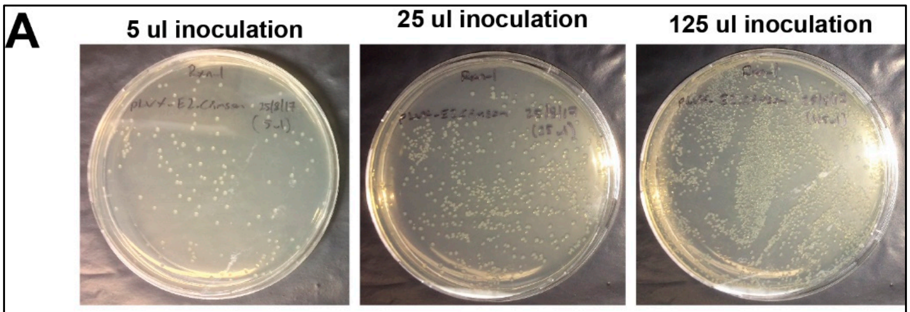

B

Bacteria inoculation volume	Clone number
5 ul	122
25 ul	611
125 ul	> 2,520

Figure S2. Heat shock technique is used to transform the competent bacteria with newly engineered pLVX-E2-Crimson-C1 lentiviral constructs. A, Efficiency of bacterial colony formation is assessed by inoculating different volumes of heat-shocked bacteria. B, Quantification of bacteria clones in A after 16 hours of incubation at 37°C.

References

1. Stringer, B.W.; Day, B.W.; D'Souza, R.C.J.; Jamieson, P.R.; Ensbey, K.S.; Bruce, Z.C.; Lim, Y.C.; Goasdoue, K.; Offenhauser, C.; Akgul, S.; et al. A reference collection of patient-derived cell line and xenograft models of proneural, classical and mesenchymal glioblastoma. *Sci Rep* **2019**, *9*, 4902, doi:10.1038/s41598-019-41277-z.
2. QIMR, B.M.R.I. QCell. Available online: https://www.qimrberghofer.edu.au/commercial-collaborations/partner-with-us/qcell/ (accessed on 14/02/2022).
